# Young adults’ BMI and changes in romantic relationship status during the first semester of college

**DOI:** 10.1371/journal.pone.0230806

**Published:** 2020-03-26

**Authors:** Irene van Woerden, Alexandra Brewis, Daniel Hruschka, Genevieve Dunton, Marc A. Adams, Meg Bruening

**Affiliations:** 1 College of Nursing, Idaho State University, Pocatello, Idaho, United States of America; 2 School of Human Evolution and Social Change, Arizona State University, Tempe, Arizona, United States of America; 3 Institute for Health Promotion & Disease Prevention, University of Southern California, Los Angeles, California, United States of America; 4 College of Health Solutions, Arizona State University, Phoenix, Arizona, United States of America; McMaster University, CANADA

## Abstract

**Purpose:**

Identify how higher Body Mass Index (BMI) and weight discrimination are associated with romantic relationship formation and termination in young adults, and if the association was consistent for males and females.

**Methods:**

First-year students (N = 1096) at entry to university (Time 1) provided BMI and self-reports of weight discrimination and romantic relationship status (in a relationship vs single); 550 were successfully resampled four months later (Time 2). Logistic generalized estimating equations (GEEs) examined if Time 1 relationship status was predicted by BMI and weight discrimination. Logistic GEEs were used to determine if Time 1 BMI and weight discrimination predicted Time 2 relationship status for the strata of students in, and out, of a relationship at Time 1.

**Results:**

At baseline, students were less likely to be in a relationship if they had a higher BMI (OR = 0.94, 95% CI = 0.92, 0.96) or reported weight discrimination (OR = 0.69, 95% CI = 0.53, 0.90). When stratified by gender, the association between higher BMI and weight discrimination with relationship status was only observed for females. Longitudinally, a BMI-based selection effect was observed for romantic relationship formation, but not termination. Of the students who were single at Time 1, each one unit higher baseline BMI decreased the odds of the student transitioning to a relationship by 9% at Time 2 (OR = 0.91, 95% CI = 0.85, 0.96). When stratified by gender the association of higher BMI decreased odds of relationship formation was only significant for females. No weight discrimination differences for selection in or out of a romantic relationship were observed.

**Conclusions:**

These findings suggest a weight-related selection effect for romantic relationship initiation, but not termination, in young female adults with lower BMIs. Weight discrimination was not associated with romantic relationship initiation or termination in this sample.

## Introduction

Body size can shape social experiences and prospects, including romantic relationships [1–4]. Studies of large-scale marital data suggest that slimmer women are viewed as more desirable romantic partners and are more likely to marry [4–7]. Research also suggests that adolescents and young adults with an overweight weight status are less likely to be married seven years later [4]. It is less clear how weight affects entry into (and exit from) relationships in adolescence and early adulthood prior to marriage decisions. First-year college students living together in residence halls are an excellent sentinel group for such a study; the opportunity for initiating new romantic and sexual relationships can be high for these students [8–11].

Gender differences with higher Body Mass Index (BMI) and relationships occur. Studies indicate female undergraduate students with an overweight weight status (BMI ≥25) are less likely to be seen as a desirable partner [2] or to be in a relationship [12] than their female counterparts without an overweight weight status. Female university students with an overweight weight status are reported to date less [12] and research among adolescent females shows similar findings [13]. In contrast, one study concluded that there was no difference in BMI by undergraduate male college students who were, and were not, dating [12]. Another study asked undergraduate students to rank the desirability of a range of hypothetical potential sexual partners, both students with and without an overweight weight status stated that their lowest preference was for a person with obesity [14]; male students considered a potential partner with obesity as even less desirable than female students did [14].

While college student (and other young adult) weight status has been shown to be associated with relationship *initiation*, it remains less clear if weight status also influences relationship *termination*. This matters, because if weight influences relationship termination, it suggests an additional possible mechanism of weight stigma and discrimination faced by young adults [2, 15], and there is a growing body of evidence that shows the experience of stigma-related rejection around weight is extremely stressful and can have profound negative effects on health [16].

The purpose of the current study is to examine first-year students’ weight, weight discrimination, and romantic relationship status. Given previous research [12, 14] we also examine differences by gender. We check the hypothesis that first year students with a higher BMI will be less likely to be in a relationship than their counterparts with a lower BMI. We also hypothesize that students who exhibit weight discrimination will be less likely to be in a relationship. We hypothesize that limiting potential dating pools to those of a certain weight status results in a smaller dating pool, and a lower probability of finding a suitable partner. We additionally hypothesize that weight discrimination is an unattractive trait in a potential partner, and that exhibiting weight discrimination further limits the potential dating pool. We then test if relationship status change is associated with baseline BMI and weight discrimination. We hypothesize that first year college students with a higher BMI will be less likely to enter into, and remain in, a relationship compared to their lower BMI peers. We also hypothesize that participants who exhibit weight discrimination will be less likely to enter into a relationship given the smaller dating pool and as discriminating based on weight is potentially an unattractive trait.

## Methods

### Data source

This secondary analysis used existing data from the larger SPARC (Social Impact of Physical Activity and Nutrition in College) study. The main aim of the SPARC study was to determine how friendship networks were associated with change in first-year college students’ eating and physical activity behaviors and weight gain [17]. Recruitment was primarily through the residence hall floor meetings at the start of the Fall 2015 semester (Time 1) however a few students were recruited through peer-referral. Student follow-up occurred at the same residence halls at the end of the Fall 2015 semester (Time 2). Relationship status was examined at the start and end of the Fall semester (four months apart) so as to capture relationship changes during the period when students were new to campus and (theoretically) meeting each other for the first time. As relationship initiation and formation may occur differently over holiday periods, relationship status was not examined between semesters. All students provided written informed consent. All study protocols were approved by the Arizona State University Institutional Review Board.

### Sample and design

At Time 1, 1096 first-year students completed the first survey and had their BMI measured by trained research assistants. Of those students who completed the first survey, 50% (n = 550) reported their relationship status at Time 2. Retention was low in part due to issues with the technology involved in another aspect of the study. Compared to the students who only completed the Time 1 survey, the students who completed the Time 2 survey were more likely to be female (58.6% vs 72.4%, P<0.001), to have a higher BMI (23.59 vs 24.20, P = 0.039), and to not report weight discrimination (33.0% vs 43.1%, P = 0.001; Table 1).

**Table 1:** Comparison of the demographics and key variables participants who were, and were not, in the longitudinal dataset.

	Cross-sectional only	Longitudinal	P.value
n	546	550	
**Gender, n (%)**			**<0.001**
Female	320 (58.6)	398 (72.4)	
Male	226 (41.4)	152 (27.6)	
**Race/ethnicity, n (%)**			0.249
Non-Hispanic White	291 (53.3)	273 (49.6)	
Other	255 (46.7)	277 (50.4)	
**Pell Grant status, n (%)**			0.128
No	378 (69.2)	356 (64.7)	
Yes	168 (30.8)	194 (35.3)	
**BMI, mean (SD)**	23.6 (4.6)	24.2 (5.0)	**0.039**
**Weight status, n (%)**			**0.007**
Under weight	40 (7.3)	21 (3.8)	
Normal weight	351 (64.3)	337 (61.3)	
Over weight	101 (18.5)	139 (25.3)	
Obese	54 (9.9)	53 (9.6)	
**Weight discrimination, n (%)**			**0.001**
No	180 (33.0)	237 (43.1)	
Yes	366 (67.0)	313 (56.9)	
**Relationship status, n (%)**			0.275
Single	384 (70.3)	369 (67.1)	
In a relationship	162 (29.7)	181 (32.9)	
**Relationship duration, n(%)**^A^			1.000
One year or less	71 (52.6)	88 (52.1)	
More than one year	64 (47.4)	81 (47.9)	
**Relationship time in person, n (%)**^A^			0.934
Less than half an hour per week	61 (37.7)	70 (38.7)	
At least half an hour per week	101 (62.3)	111 (61.3)	
**Residence Hall, n (%)**			
A	159 (29.1)	282 (51.3)	**<0.001**
B	11 (2.0)	22 (4.0)	
C	174 (31.9)	98 (17.8)	
D	24 (4.4)	30 (5.5)	
E	95 (17.4)	65 (11.8)	
F	70 (12.8)	47 (8.5)	
G	13 (2.4)	6 (1.1)	

^A^ only for the participants who were in a relationship

#### Measures

*Anthropometrics*. Trained research assistants obtained students’ height using Seca stadiometers (model 217) and weight using Seca flat scales (models 874 or 869) at Time 1 and Time 2. Body mass index (BMI) was calculated as weight/height^2^ (kg/m^2^) and centered at the BMI score of 25.

*Relationship status*. Students were asked at each time point: “How would you describe your current relationship status?”. The response options were “In a relationship” and “Single”. Students were classified as being selected into a relationship during the course of the study if they reported being single at Time 1 and in a relationship at Time 2. Conversely, students were classified as having had a relationship terminate if they were in a relationship at Time 1 and single at Time 2. The gender of the person the student was in a relationship with, and the students’ sexual orientation, were not assessed in this study. To assess relationship duration, students in a relationship were asked “how long have you been in this current relationship” with the duration reported in months. Relationship duration was categorized as one year or less vs more than one year based on the distribution of responses. Students in a relationship were also asked “how much time in a week do you see (in-person) your significant other”. The response options ranged between “less than one hour” and “40 or more hours”, due to the distribution of responses time spent in-person with significant other was dichotomized to less than one hour vs more than one hour.

*Weight discrimination*. To gauge weight discrimination at Time 1 an adapted question from Bogardus was used. Participants were asked “Would you hesitate to have a romantic relationship with a person who is obese?” [18]. The response options were on a four-point agree/disagree scale and dichotomized to agree (yes) vs disagree (no).

*Sociodemographic characteristics*. Information on students’ gender, age, family income, race/ethnicity, and living location were obtained. Age was determined from the students’ date of birth. Family income was determined by asking if students were Pell Grant recipients (a Pell Grant is a federal grant provided to low income students). Students were asked “How do you usually describe yourself? (check all that apply)” with response options “White”; “Black or African American”; “Hispanic or Latino/a”; “Asian or Pacific Islander”; “American Indian or Alaska Native”; “Some Other race (please specify)”. Due to low counts for certain classifications, race/ethnicity was categorized as non-Hispanic White and Other. For this study six first-year students’ residence halls were targeted. Students’ living locations were classified to one of the six targeted residence halls, or other living location.

### Statistical analysis

The difference between the participants who were, and were not, in the longitudinal (Time 1 and Time 2) sample was examined using χ^2^ and Wilcoxon tests as appropriate. The bivariate associations of the sociodemographic factors, BMI, and weight status with relationship status at Time 1 were also examined using χ^2^ and Wilcoxon tests as appropriate. A logistic generalized estimating equation (GEE) was used to determine if Time 1 relationship status was predicted by Time 1 BMI and Time 1 weight discrimination. Controls for gender, race/ethnicity, family income status, and clustering within residence halls were included in the model. To test for gender differences, the same model but stratified by gender was run.

The association between Time 1 BMI and change in relationship status over the semester was examined using logistic GEE models. As different selection effects may occur for selection into, and out of, a relationship, students who were, and who were not, in a relationship at Time 1 were stratified and examined separately. For both strata, relationship status at Time 2 was predicted by the individual’s BMI and weight discrimination at Time 1. Controls for gender, race/ethnicity, family income status, and clustering within residence hall were included in the models. For the strata of participants who were in a relationship at Time 1, the same model but including relationship duration and time spent in person was also run. To test for gender differences, the same analyses were then re-run stratified by gender. All analyses were done using the statistical software R (version 3.6.2). Significance was determined at p<0.05.

## Results

Bivariate results from Time 1 showed that the females were more likely to be in a relationship than males (p<0.001; Table 2). The Time 1 bivariate results also indicated that individuals with a lower BMI, and individuals who did not state they would hesitate to have a relationship with someone with obesity, (i.e., admit romantic discrimination) were more likely to be in a relationship (p<0.001, p = 0.023; Table 2).

**Table 2:** Demographics and key variables at baseline.

	Total	Not in a relationship	In a relationship	P.value
n	1096	753	343	
**Gender, n (%)**				**<0.001**
Female	718 (65.5)	464 (61.6)	254 (74.1)	
Male	378 (34.5)	289 (38.4)	89 (25.9)	
**Race/ethnicity, n (%)**				0.696
Non-Hispanic White	564 (51.5)	384 (51.0)	180 (52.5)	
Other	532 (48.5)	369 (49.0)	163 (47.5)	
**Pell Grant Status, n (%)**				0.560
No	734 (67.0)	509 (67.6)	225 (65.6)	
Yes	362 (33.0)	244 (32.4)	118 (34.4)	
**BMI, mean (SD)**	23.9 (4.8)	24.3 (5.0)	23.1 (4.1)	**<0.001**
**Weight status, n (%)**				**0.025**
Underweight	61 (5.6)	36 (4.8)	25 (7.3)	
Normal weight	688 (62.8)	463 (61.5)	225 (65.6)	
Overweight	240 (21.9)	169 (22.4)	71 (20.7)	
Obese	107 (9.8)	85 (11.3)	22 (6.4)	
**Weight discrimination, n (%)**				**0.023**
No	417 (38.0)	269 (35.7)	148 (43.1)	
Yes	679 (62.0)	484 (64.3)	195 (56.9)	
**Relationship duration, n (%)**				NA
One year or less	159 (52.3)	NA	159 (52.3)	
More than one year	145 (47.7)	NA	145 (47.7)	
**Time in person, n (%)**				NA
less than half an hour	131 (38.2)	NA	131 (38.2)	
half an hour or more	212 (61.8)	NA	212 (61.8)	
**Residence Hall, n (%)**				0.593
A	441 (40.2)	296 (39.3)	145 (42.3)	
B	33 (3.0)	23 (3.1)	10 (2.9)	
C	272 (24.8)	199 (26.4)	73 (21.3)	
D	54 (4.9)	38 (5.0)	16 (4.7)	
E	160 (14.6)	109 (14.5)	51 (14.9)	
F	117 (10.7)	77 (10.2)	40 (11.7)	
G	19 (1.7)	11 (1.5)	8 (2.3)	

Bold indicates significant finding (P<0.05)

The majority of students had the same romantic relationship status at both Time 1 and Time 2. Of the students in the longitudinal sample who were not in a relationship at Time 1 (n = 369), only 51 (14%) were in a relationship at Time 2; of the 181 students who were in a relationship at Time 1, 76% (n = 138/181), were also in a relationship at Time 2.

In the cross-sectional GEE models controlling for demographics, students remained significantly less likely to be in a relationship if they were male (OR = 0.59, 95% CI = 0.46, 0.76), had a higher BMI (OR = 0.94, 95% CI = 0.92, 0.96), or reported weight discrimination (OR = 0.69, 95% CI = 0.53, 0.90; Table 3). When stratified by gender the odds ratio for BMI and weight discrimination were similar (OR = 0.94 and 0.95 vs 0.94; OR = 0.70 and 0.66 vs 0.69) however the effects were only statistically significant for females.

**Table 3:** Cross-sectional generalized estimating equation models predicting relationship status^a^ by demographics, BMI, and weight stigma (n = 1096).

	All (n = 1096)			Female (n = 718)			Male (n = 378)		
	OR	95% CI	P.value	OR	95% CI	P.value	OR	95% CI	P.value
**Gender**									
Female	(ref)								
Male	0.59	(0.46, 0.76)	**<0.001**						
**Race/ethnicity**									
Non-Hispanic White	(ref)			(ref)			(ref)		
Other	0.89	(0.66, 1.19)	0.438	1.16	(0.79, 1.71)	0.443	0.45	(0.25, 0.83)	**0.011**
**Pell Grant Status**									
No	(ref)			(ref)			(ref)		
Yes	1.18	(0.93, 1.49)	0.168	1.13	(0.84, 1.54)	0.417	1.27	(0.79, 2.05)	0.320
**BMI**	0.94	(0.92, 0.96)	**<0.001**	0.94	(0.91, 0.97)	**<0.001**	0.95	(0.90, 1.00)	0.052
**Weight discrimination**									
No	(ref)			(ref)			(ref)		
Yes	0.69	(0.53, 0.90)	**0.007**	0.70	(0.53, 0.95)	**0.020**	0.66	(0.37, 1.16)	0.149

^a^ Relationship Status: 1 = in a relationship, 0 = not in a relationship

Bold indicates significant finding (P<0.05)

Students who were not in a relationship at Time 1 (n = 369) were 9% less likely to be in a relationship at Time 2 for each one-unit higher Time 1 BMI (OR = 0.91, 95% CI = 0.85, 0.96; Table 4). No association with weight discrimination and relationship initiation was found (OR = 1.27, 95% CI = 0.69, 2.35). Once stratified by gender the effect of BMI was only significant for females (Female OR = 0.86, 95% CI = 0.78, 0.95; Male OR = 0.98, 95% CI = 0.90, 1.07). A lack of association remained for weight discrimination and relationship initiation when stratified by gender.

**Table 4:** Longitudinal models examining the effect of BMI and weight discrimination on Time 2 relationship initiation^a^.

	All (n = 369)			Female (n = 259)			Male (n = 110)		
	OR	95% CI	P.value	OR	95% CI	P.value	OR	95% CI	P.value
**Gender**									
Female	(ref)								
Male	0.92	(0.53, 1.59)	0.764						
**Race/ethnicity**									
Non-Hispanic White	(ref)			(ref)			(ref)		
Other	0.66	(0.36, 1.21)	0.181	0.64	(0.35, 1.16)	0.139	0.65	(0.19, 2.14)	0.474
**Pell Grant Status**									
No	(ref)			(ref)			(ref)		
Yes	0.64	(0.31, 1.32)	0.225	0.66	(0.30, 1.44)	0.293	0.65	(0.14, 3.00)	0.577
**BMI**	0.91	(0.85, 0.96)	**0.001**	0.86	(0.78, 0.95)	**0.003**	0.98	(0.90, 1.07)	0.677
**Weight Discrimination**									
No	(ref)			(ref)			(ref)		
Yes	1.27	(0.69, 2.35)	0.438	1.57	(0.74, 3.34)	0.243	0.76	(0.24, 2.42)	0.637

^a^ Relationship Status: 1 = in a relationship, 0 = not in a relationship

Bold indicates significant finding (P<0.05)

Of the students who were in a relationship at Time 1 (n = 181), the odds of a student being in a relationship at Time 2 was not associated with their Time 1 BMI (OR = 0.96, 95% CI = 0.89, 1.02) or weight discrimination (OR = 0.73, 95% CI = 0.38, 1.42; Table 5). No association with BMI or weight discrimination and relationship termination was found for the female strata (male strata not run due to small sample size, n = 42).

**Table 5:** Longitudinal models examining the effect of BMI and weight stigma on Time 2 relationship termination^a^.

	All (n = 181)			Female (n = 139)^b^		
	OR	95% CI	P.value	OR	95% CI	P.value
**Gender**						
Female	(ref)					
Male	1.46	(0.62, 3.4)	0.383			
**Race/ethnicity**						
Non-Hispanic White	(ref)			(ref)		
Other	0.75	(0.38, 1.48)	0.411	1.13	(0.53, 2.41)	0.751
**Pell Grant Status**						
No	(ref)			(ref)		
Yes	1.37	(0.71, 2.64)	0.353	1.57	(0.72, 3.39)	0.253
**BMI**	0.96	(0.89, 1.02)	0.176	0.98	(0.91, 1.06)	0.618
**Weight Discrimination**						
No	(ref)			(ref)		
Yes	0.73	(0.38, 1.42)	0.359	0.58	(0.29, 1.15)	0.119

^a^ Relationship Status: 1 = in a relationship, 0 = not in a relationship

^b^ The stratification for male was not run given the small sample size (n = 42)

When examining measures of relationship quality, neither relationship duration (OR = 1.36, 95% CI = 0.63, 2.96), nor time spent in person (OR = 0.67, 95% CI = 0.32, 1.40) were associated with relationship termination (Table 6). These results remained non-significant for the female strata (male strata not run due to small sample size, n = 42).

**Table 6:** Longitudinal models examining the effect of BMI and weight stigma on Time 2 relationship termination^a^.

	All (n = 169)			Female (n = 129)^b^		
	OR	95% CI	P.value	OR	95% CI	P.value
**Gender**						
Female	(ref)					
Male	1.45	(0.54, 3.89)	0.459			
**Race/ethnicity**						
Non-Hispanic White	(ref)			(ref)		
Other	0.73	(0.36, 1.47)	0.375	1.21	(0.55, 2.63)	0.638
**Pell Grant Status**						
No	(ref)			(ref)		
Yes	1.55	(0.77, 3.11)	0.217	1.94	(0.76, 4.94)	0.164
**BMI**	0.96	(0.90, 1.03)	0.273	1.00	(0.93, 1.08)	0.977
**Weight Discrimination**						
No	(ref)			(ref)		
Yes	0.68	(0.36, 1.29)	0.237	0.53	(0.26, 1.08)	0.082
**Relationship Duration, n (%)**						
One year or less	(ref)			(ref)		
More than one year	1.36	(0.63, 2.96)	0.433	1.87	(0.80, 4.38)	0.151
**Time in person, n (%)**						
less than half an hour	(ref)			(ref)		
half an hour or more	0.67	(0.32, 1.40)	0.285	0.55	(0.260, 1.16)	0.118

^a^ Relationship Status: 1 = in a relationship, 0 = not in a relationship

^b^ The stratification for male was not run given the small sample size (n = 42)

## Discussion

This study examined the association between relationship status, BMI, and weight discrimination over a period of four months in which young adults had heightened opportunity for forming new romantic relationships (the first semester of college). At Time 1, first year students with a lower BMI, who did not report weight discrimination, and who were female were more likely to be in a relationship than their counterparts. Higher BMI was associated with lack of relationship initiation (for females), but not relationship termination. Weight discrimination was not associated with relationship initiation or termination. The two measures of relationship quality, relationship duration and time spent in person, were not associated with relationship termination.

Cross-sectionally, students who reported weight discrimination were less likely to be in a relationship than students who did not report weight discrimination. However, weight discrimination was not associated with relationship formation or termination. This may suggest that weight discrimination changes with relationship status, with students not in a relationship more likely to report weight discrimination than students in a relationship. Another possibility is that the number of potential relationships is much larger once students’ start college, increasing the size of students’ dating pool. Prior to college (with a small dating pool) weight discrimination may have limited students’ potential relationships. However when the number of potential relationships is large (such as in the college environment) limiting relationships to those of a specific weight may have a negligible effect on relationship options.

Of the students who were not in a romantic relationship at Time 1, those with lower BMI values at Time 1 were more likely to enter into a romantic relationship at Time 2. These findings suggest the possibility of a systematic exclusionary bias against initiating a relationship with a higher BMI first year student. Students with higher BMI may be being excluded from romantic relationships. Another possibility is that people with higher body weights may elect to avoid forming romantic relationships [19]. However, we have been unable to locate any literature providing evidence of this possibility; by contrast there are longitudinal studies showing rejection as a basis for non-formation of desired friendships (e.g., Simpkins et al., 2013 [20]). In particular, university is a period of identity formation [21] and students may be intentionally choosing to remain single. Additionally, if the students with higher BMI are in the process of losing weight, which can be a period of self-focus [22], focusing on a relationship may be a low priority.

Time 1 BMI was not associated with relationship termination across the semester. This finding is congruent with previous studies identifying that BMI was not a significant predictor of divorce risk [23, 24]. However, in this longitudinal analyses, the majority of the students were not in a relationship at Time 1, which significantly reduced the number of observations available on students who ended a relationship, and should be considered a limitation of the study findings. Notably, the odds ratio for BMI for relationship termination and initiation were similar (OR = 0.91, 0.96), suggesting the “non-significant” results for relationship termination may be due to the smaller sample size. While no association with relationship termination and BMI when using the blunt measure of “in” or “out” of a relationship was observed we suggest further research is needed to confirm or refute this.

It has been shown in U.S.-based studies that females experience more weight discrimination than males [25, 26]. We observed a gendered effect on BMI for relationship initiation. Our prior ethnographic work with college students on the same campus shows that many younger male students are now sensitive to weight judgments in ways that are similar to female peers—although males also worry more about being underweight [27]. Another recent study indicated that males are increasingly reporting weight stigma [28]. The findings from this research may indicate that female young adult college students with an overweight weight status are still facing lowered opportunities or heightened distancing from relationship initiation when compared to their male counterparts.

More females reported being in a relationship at Time 1 (35%,254/718) than males (24%, 89/378). A limitation of this study is that students were asked if they were “in a relationship” or “single” but a definition of “in a relationship” was not provided. Notably, the interpretation of “in a relationship” may be different by gender. For instance one study suggests that young women are more likely to perceive a casual sex (i.e. sex outside of a relationship) encounter as the beginning of a relationship, while young men were more likely to perceive a casual sex encounter as the beginning of a subsequent casual sex relationship [9]. Studies indicate that casual sex and “hooking up” are common among undergraduate students [8–11], which by some social definitions [29] does not equate to a “relationship.” As how students interpreted the definition of being “in a relationship” and “single” is unclear, despite asking the questions using these terms, the gender difference observed may partially be due to gender differences in the interpretation of the question.

Another limitation of the study is the short duration (four months) of the study period and the lack of detailed information on relationship status. While the time spent in person with the romantic partner and relationship duration were collected, the quality of relationship was unknown. It was unclear if students were in a relationship with someone at the same university or not, the gender of the person they were in a relationship with, and the sexual orientation of the participant. The number and duration of students’ prior relationships was unknown. Given the sample size, relationship duration was only examined at the dichotomous level of more than one year vs not. Relationship status was only examined at the start and end of the semester. Students who began, and ended, a relationship during the first semester was classified into the same category as someone who was never in a relationship in the first semester. It was unclear if the students who were in a relationship at Time 1 and Time 2 were in a relationship with the same person, if the relationship had been continuous between the two time periods, and if students not in a relationship at the start of the semester were only recently single. More frequent measurements which also examine the quality of the relationship and rate of partner change should be examined, along with partner information. In particular, partner weight status was not collected in this study which limits the extent of dyadic research from this study.

Other study limitations should be considered when interpreting findings. This study uses only one measure (hesitation to have a romantic relationship with a person who is obese) to determine which individuals exhibited weight discrimination. The sample size for the longitudinal sample was small. Systematic differences in unmeasured variables for the students’ who were, and were not, lost to follow up may have biased the results. It was unclear which students had classes together which may have affected relationship formation; however, students were assigned to residence halls based on major (controlled for in the models). The lack of significant findings for males may be due to a lack of power to detect these effects, and future suitably powered studies may shed further light on the role that weight judgments play in relationship formation for all genders. Lastly, these findings are from first year students from a specific university, it is unclear if these results will generalize to other universities and to students not in their first year.

## Conclusion

In this sample of young college-living adults, higher BMI, male gender, and weight discrimination was associated with lower likelihood of being in a romantic relationship. Romantic relationship initiation was associated with lower BMI for females but not males. Lower BMI was not associated with relationship termination and weight discrimination was not associated with relationship initiation or termination. This adds additional evidence that BMI shapes selection into a relationship for young females but not young males, and that BMI is not associated with relationship maintenance/termination.

## Supporting information

S1 Dataset (CSV)
